# The role of the interleukin family in liver fibrosis

**DOI:** 10.3389/fimmu.2025.1497095

**Published:** 2025-02-10

**Authors:** Zixin Zhang, Jiahui Wang, Hui Li, Qun Niu, Yujing Tao, Xin Zhao, Zijian Zeng, Haijian Dong

**Affiliations:** ^1^ Central Laboratory, Hospital of Chengdu University of Traditional Chinese Medicine, Chengdu, China; ^2^ School of Clinical Medicine, Chengdu University of Traditional Chinese Medicine, Chengdu, China

**Keywords:** cytokines, interleukins, interleukin receptors, inflammation, liver fibrosis

## Abstract

Liver fibrosis represents a wound-healing response to chronic liver injury caused by viral infections, alcohol, and chemicals agents. It is a critical step in the progression from chronic liver disease to cirrhosis and hepatocellular carcinoma. No chemical or biological drugs have been approved for the treatment of liver fibrosis. Relevant studies have demonstrated that effective inhibition of hepatitis B virus (HBV) replication by nucleoside (acid) analogs or polyethylene glycol alpha-interferon can lead to recovery in some patients with hepatitis B liver fibrosis, However, some patients with liver fibrosis do not show improvement, even after achieving a complete serologic and virologic response. A similar situation occurs in patients with hepatitis C-related liver fibrosis. The liver, with its unique anatomical and immunological structure, is the largest immune organ and produces a large number of cytokines in response to external stimuli, which are crucial for the progression of liver fibrosis. cytokines can act either by directly affecting hepatic stellate cells (HSCs) or by indirectly regulating immune target cells. Among these, the interleukin family activates a complex cascade of responses, including cytokines, chemokines, adhesion molecules, and lipid mediators, playing a key role in the initiation and regulation of inflammation, as well as innate and adaptive immunity. In this paper, we systematically summarize recent literature to elucidate the pathogenesis of interleukin-mediated liver fibrosis and explore potential therapeutic targets for liver fibrosis treatment.

## Introduction

Liver fibrosis is a pathological change that occurs during the course of most chronic liver diseases. It is caused by chronic inflammation due to the persistent effects of various etiologies such as viral hepatitis infections, non-alcoholic fatty liver disease, autoimmune liver disease, and drug-induced liver injury. This inflammation leads to liver damage and, consequently, to liver fibrosis (1, 2). Currently, no chemical or biological drugs have been approved for the treatment of liver fibrosis (3). In modern medicine, the treatment of liver fibrosis is primarily etiological. Although studies have demonstrated that liver fibrosis is a reversible pathological process (4), without early intervention, it can progress to cirrhosis or even hepatocellular carcinoma. Approximately more than 2 million people die each year from chronic liver disease (5).

The primary pathological feature of liver fibrosis is the excessive pathological proliferation and deposition of extracellular matrix (ECM) (2). HSCs, the main fibrotic cell type, reside in the perisinusoidal space between hepatic sinusoidal endothelial cells and hepatocytes (6). These cells are non-substantial hepatocytes, accounting for about 15% of the total number of resident cells (7). In the normal liver, HSCs maintain a nonproliferative, quiescent phenotype. However, during liver injury, hepatocytes undergo apoptosis or necrosis, releasing proinflammatory and profibrotic cytokines. These cytokines stimulate the recruitment and activation of inflammatory cells in the liver. At this point, HSCs became activated and transform from vitamin A-storing cells into myofibroblasts with proliferative, contractile, and chemotactic functions. These activated HSCs produce excessive ECM components that accumulate in liver parenchymal cells, disrupting liver structure and forming characteristic scar tissue (8). Activation of HSCs is central to the pathogenesis of liver fibrosis, and HSCs are the primary cellular source of ECM production (7). A variety of cytokines act as “messenger” proteins involved in regulating immunity, cell growth, and tissue repair through paracrine and autocrine effects on target cell-specific receptors. Several cytokines regulate liver fibrosis, particularly through the regulation of collagen metabolism in HSCs and intercellular matrix.

Cytokines include interleukins, interferons, growth factors, chemokines. Among them, the role of the interleukin family in liver fibrosis has gained increasing attention. There are 33 known interleukins, along with numerous derivatives such as Interleukin-1 beta (IL-1β), Interleukin-36 alpha (IL-36α), and Interleukin-36 gamma (IL-36γ). Interleukins are primarily secreted by various of immune cells (e.g., macrophages, neutrophils), and are glycoproteins that act on a variety of cells throughout the body. They regulate immune cell activation, proliferation, secretion, and other processes,playing an essential role in the progression of the inflammatory response. Numerous studies have shown that the interleukin family plays a critical role in the development of liver fibrosis. On one hand, pro-inflammatory interleukins such as Interleukin-1 (IL-1), Interleukin-6 (IL-6), Interleukin-17 (IL-17), Interleukin-18 (IL-18), Interleukin-33 (IL-33), and Interleukin-36 (IL-36) enhance tissue damage and inflammation. On the other hand, anti-inflammatory interleukins such as Interleukin-10 (IL-10), Interleukin-35 (IL-35), and Interleukin-37 (IL-37) promote tissue regeneration and play a protective role in the liver. Additionally, Interleukin-4 (IL-4), Interleukin-22 (IL-22) exerts both anti-inflammatory and pro-inflammatory roles in liver fibrosis.

Currently, interleukins are widely recognized for their regulatory roles in the development of liver fibrosis. However, the overall regulatory mechanisms remain unclear, and the specific signaling pathways and transduction links involved need further investigated. Therefore, in this article, we systematically summarize recent literature to elucidate the pathogenesis of interleukin-family-mediated liver fibrosis and review fibrosis drugs currently targeting interleukins as therapeutic candidates. We aim to provide new insights into the treatment of liver fibrosis.

## Interleukins exerting pro-liver fibrosis effects

### Interleukin-1

IL-1 is one of the earliest cytokines discovered.It is mainly produced by monocytes, macrophages, T lymphocytes (T cells), B lymphocytes (B cells) and Natural Killer cells (NK cells) (9). Additionally, almost all nucleated cells can produce IL-1. In the liver, it is primarily expressed by Kupffer cells (KCs), hepatic endothelial cells, and HSCs, mainly in the form of Interleukin-1 alpha (IL-1α) and IL-1β, both forms recognize a common receptor, Interleukin-1 Receptor (IL-1R), which consists of the subunits IL-1R1 and Interleukin-1 Receptor Accessory Protein (IL-1RacP). These receptors mediate IL-1’s involvement in immune responses, inflammation, and fibrogenesis (10). IL-1 is synthesized as a precursor, pro-IL-1, which is converted to its active form. The active cytokines promotes fibrosis by being released extracellularly, thereby initiating a cascade of inflammatory responses in target cells, such as HSCs (11). Among the two isoforms, IL-1α is a bifunctional cytokine. Extracellularly, IL-1α binds to IL-1R1 on the cell surface and recruits its co-receptor IL-1R3, initiating pro-inflammatory signaling similar to IL-1β. Intracellularly, IL-1α can shuttle rapidly between the nucleus and cytoplasm, enabling distinct biological functions in different cellular compartments. Since there is limited research on the role of IL-1α in liver fibrosis, this review primarily focuses on IL-1β, an inducible and highly inflammatory cytokine.IL-1β activates a complex signaling cascade via IL-1R1, which subsequently triggers transcription factors such as nuclear factor-kappa B (NF-κB) and induces the production of inflammatory cytokines (12). It has been shown that IL-1β contributes to fibrosis through the MyD88-IRAK-NF-κB signaling pathway by forming a tripartite complex with IL-1R1 and IL-1RacP (13–15).

In patients with chronic liver disease, serum expression of IL-1β is elevated, and IL-1β has been shown implicated of hepatic steatosis to steatohepatitis and hepatic fibrosis (16, 17).Hepatic tissue expression of IL-1α and IL-β is significantly increased in diet-induced nonalcoholic steatohepatitis (NASH) models, indicating that these cytokines are involved in the regulation of steatosis and steatohepatitis. Despite a reduced inflammatory response, both liver cholesterol and serum cholesterol levels were elevated in IL-1α-deficient mice, suggesting that IL-1α may influence hepatic fat accumulation and inflammation through distinct pathways. However, studies using IL-1α and IL- 1β knockout mice showed improved diet-induced steatosis, indicating that both IL-1α and IL- 1β contribute to NASH development (18). Furthermore, liver and non-bone marrow-derived IL-1α/β deficiency ameliorated diet-induced liver inflammation and fibrosis, suggesting a critical role for liver-derived IL-1 in this context (18). High expression of the immune checkpoint T-cell Immunoglobulin and Mucin-domain containing-3 (Tim-3) on hepatic macrophages has been shown to attenuates inflammation-related hepatic injury in a NASH mouse model. *In vitro* assays demonstrated that Tim-3 negatively regulates reactive oxygen species production and secretion of pro-inflammatory cytokines, such as IL-1β, in macrophages, thereby reducing the severity of inflammatory injury in NASH (19). In mice with high-fat diet-induced nonalcoholic fatty liver disease (NAFLD), upregulation of IL-1β in hepatocytes contributes to increased fat aggregation, liver inflammation, insulin resistance and liver fibrosis (20). KCs play an important role in liver injury in alcohol-fed models. Acute and chronic alcohol feeding activates KCs via the “lipopolysaccharide-toll-like receptor 4” signaling axis (21), leading to the production of pro-inflammatory mediators such as IL-1β and Tumor Necrosis Factor alpha (TNF-α). This cascade ultimately results in hepatocellular dysfunction, apoptosis, necrosis, and the ECM production by HSCs, contributes to liver fibrosis and cirrhosis (22). In alcoholic liver injury models using IL-1β-deficient mice, liver damage was significantly reduced, highlighting the regulator role of the IL-1β signaling pathway is steatosis, inflammation, and liver fibrosis (22). In a rat model of chronic alcoholic liver disease, elevated IL-1β exacerbated hepatocyte injury, promoted the release of pro-fibrotic factors such as Transforming Growth Factor beta (TGF-β) and platelet-derived growth factor, and activated HSCs. These findings underscore the crucial role of IL-1β in alcoholic liver injury and its progression fibrosis, suggesting that targeting the IL-1β signaling pathway could offer therapeutic potential (18).

Caspase-1-mediated pyroptosis is a classical mechanism that induces the maturation of inflammatory cytokines, such as IL-1β, triggering cell lysis and death, promoting pyroptosis, activating inflammasomes, and driving fibrosis development (23). Experimental studies have shown that KCs activation by Lipopolysaccharide releases pro-inflammatory cytokines and fibrogenic factors, inducing hepatocyte pyroptosis, HSCs activation, and ECM production (24); The NOD-like Receptor Pyrin domain-containing 3 (NLRP3) inflammasome has been found to induced IL-1β secretion in primary HSCs or HSCs lines(e.g., LX-2 or HSC-T6) treated with exogenous stimulants such as MSU or bacterial RNA (25, 26). IL-1β interacts with receptors on HSCs membranes to activate NF-kB, leading to the production of Alpha-Smooth Muscle Actin (α-SMA) and type I collagen, both markers of fibrosis (25, 26).

Currently, IL-1β is being explored as a potential therapeutic target for liver fibrosis. The regulation of the Peroxisome Proliferator-Activated Receptor (PPAR) nuclear receptor family plays an important role in NASH treatment. Peroxisome Proliferator-Activated Receptor gamma (PPARγ) antagonists have demonstrated therapeutic efficacy in NASH, while Peroxisome Proliferator-Activated Receptor Delta (PPARδ) activation improved fatty acid oxidation and inhibited hepatic liposynthesis and gluconeogenesis (27). PPARδ antagonists also alleviated liver inflammation and fibrosis by inhibiting the production of pro-inflammatory factors such as IL-1β. MCC950 an inhibitor of NLRP3 inflammasome activation, downregulated IL-1β expression and significantly reduced hepatic fibrosis (28). Additionally, chuanxiongzine has been shown to protect against hepatic injury by modulating the NLRP3 inflammasome pathway, lowering IL-1β levels, and reducing fibrosis-related inflammation (29). Polysaccharides extracted from Angelica sinensis root attenuated hepatic fibrosis by inhibiting IL-1β secretion and HSCs activation (30, 31). *In vivo* and *in vivo* studies revealed that Aspergillus extracts ameliorated carbon tetrachloride (CCl4)-induced liver fibrosis and TGF-β-induced HSCs activation, likely through the Nrf2-mediated inhibition of the Reactive Oxygen Species/NOD-like Receptor Pyrin domain-containing 3/Interleukin-1 alpha (ROS/NLRP3/IL-1β) signaling pathway (32). Collectively, these studies highlight the therapeutic potential of targeting IL-1β in liver fibrosis.

### Interleukin-6

IL-6, initially identified as B-cell growth/stimulating factor II (33), is produced by various cells types, including fibroblasts, hepatocytes, monocytes/macrophages, T-cells, and endothelial cells (34). In the liver, IL-6 is predominantly expressed in hepatocytes, KCs, and HSCs. IL-6 is a pro-inflammatory cytokines with pleiotropic biological activities. Numerous studies have demonstrated that, in addition to promoting inflammatory responses, IL-6 induces lymphocyte differentiation and proliferation, facilitates HSCs activation, and contributes to the development of liver fibrosis (35).

As a pro-inflammatory cytokines, IL-6 participates in liver inflammatory responses and plays a critical role in liver fibrosis progression by regulating the secretion of pro-fibrotic factors, activating signaling pathway, and promoting the proliferation and differentiation of fibroblasts. In the transformation of chronic hepatitis B (CHB)-associated liver fibrosis to cirrhosis, significantly elevated levels of IL-6 mRNA have been detected in liver tissues, peripheral blood mononuclear cells, and serum of patients with cirrhosis. Correspondingly, IL-6 protein levels are markedly higher in cirrhosis patients compared to those with liver fibrosis (36). A positive correlation between serum IL-6 levels and the degree of fibrosis in NASH has also been established (37). Additionally, IL-6 levels were observed to increase progressively in patients with varying severities of NAFLD. These levels were significantly associated with liver enzymes, steatosis, and fibrosis, indicating that elevated peripheral serum IL-6 may promote the progression of liver fibrosis to cirrhosis (38).

Studies have demonstrated that serum IL-6 expression increases before hepatocyte necrosis occurs. Additionally, cytokines such as TGF-β, IL-6, and Interleukin-8 (IL-8) are significantly elevated in the perihepatic sinusoidal wall and interlobular septa of the liver, correlating with the degree of inflammation and liver fibrosis (39, 40). In one study (41), IL-6 gene knockout mice subjected to acute and chronic liver injury models induced by CCl4 exhibited increased α-SMA levels in HSCs, indicating the role of IL-6 in fibrosis. Glycoprotein 130 (gp130), the signal transduction receptor subunit for IL-6, plays a critical role in this pathway. In high-fat diet-induced fatty liver disease models, IL-6 or gp130 gene knockout resulted in significant improvement in liver inflammation and steatosis, as well as varying degrees of inhibition of tissue remodeling and fibrosis (42). However, in a CCl4-induced chronic liver injury model, selective gp130 gene knockout in non-parenchymal liver cells aggravated liver fibrosis, highlighting the complex role of gp130 signaling in fibrosis (43).

Mice deficient in acetaldehyde dehydrogenase and fed ethanol were more susceptible to liver inflammation and fibrosis, likely due to higher levels of malondialdehyde-acetaldehyde adducts. These adducts activate the Interleukin-6/Signal Transducer and Activator of Transcription 3 (IL-6/STAT3) pathway in the liver (44). The Extracellular Signal-Regulated Kinase (ERK) pathway also plays a significant role. ERK, a serine/threonine protein kinase with two subtypes (ERK1 and ERK2), is activated by IL-6. IL-6 synergizes with acetaldehyde or malondialdehyde to activate the Mitogen-Activated Protein Kinase (MAPK) cascade through gp130, thereby phosphorylating and initiating the ERK1/2 signaling pathway. This activation promotes HSCs activation and liver fibrosis formation in mice (45, 46). Additionally, IL-6 induces HSCs activation and liver fibrosis via the Janus Kinase/Signal Transducer and Activator of Transcription 3 (JAK/STAT3) signaling pathway (47).

Interestingly, studies have reported conflicting results regarding IL-6’s role in liver fibrosis. For instance, in high-fat diet-fed mice with myeloid-specific IL-6 receptor A knockout (IL-6RA-KO), liver fibrosis was more severe compared to wild-type mice. This phenomenon was linked to decreased levels of anti-fibrotic microRNA-223 (miR-223). IL-6 treatment was shown to prompt macrophages to release miR-223-rich exosomes, which subsequently reduced the expression of the fibrosis-promoting transcriptional coactivator with PDZ-binding motif (TAZ) in hepatocytes, thereby ameliorating liver fibrosis (48). Moreover, myeloid-specific IL-6Rα knockout mice (Il6raMye-/-) and IL-6-silenced mice subjected to high-fat diets exhibited reduced inflammation but increased liver fibrosis, further highlighting the complex and sometimes contradictory role of IL-6 in fibrosis (48). These discrepancies may stem from differences in experimental models, the broad expression of transmembrane IL-6Rα and its signaling chain gp130, and the involvement of soluble IL-6R and soluble gp130 (49).

IL-6 plays an important role in liver fibrosis caused by various etiologies. As a key pathway in fibrosis development, the IL-6-related signaling cascade holds promise as a novel serum marker for evaluating the severity of liver fibrosis. Furthermore, antibodies-based therapies targeting the IL-6 pathway represent a potential new approach for treating liver fibrosis.

### Interleukin-17

IL-17 is a characteristic cytokines of T helper 17 cells (Th17 cells), predominantly produced by Th17 cells and primarily expressed in Th17 cells, hepatocytes, and KCs in the liver (50). Interleukin-17A (IL-17A), a member of the IL-17 family, is commonly regarded as an important inflammatory mediator. It promotes the progression of liver fibrosis by acting on HSCs and contributing to ECM remodeling (51).

IL-17A has been shown to stimulate the activation of HSCs and contribute to liver fibrosis by increasing the expression of pro-inflammatory cytokines, such as IL-6, IL-1β, and TNF-α, as well as pro-fibrotic factors like TGF-β and α-SMA. The synergistic effects of IL-17A and TGF-β cytokines further activate HSCs, leading to increased collagen production and exacerbating liver fibrosis (52, 53). In a CCl4-induced mouse model of liver fibrosis, exosome-mediated activation of Toll-like Receptor 3 in HSCs during the early stages of liver injury promoted the progression of liver fibrosis by enhancing the production of IL-17A from Gamma Delta T cells (54). KCs, expressing IL-17RA and IL-17RC are activated by IL-17A, further promoting liver inflammation and fibrosis by secreting pro-inflammatory mediators and pro-fibrotic cytokines such as TGF-β, which induce HSCs activation (53, 55). *In vitro* studies have demonstrated that IL-17A induces the expression of Matrix Metalloproteinase-2 and Matrix Metalloproteinase-9 (56), and another study found that liver fibrosis was significantly reduced in a model of IL-17A receptor-deficient mice infected with Schistosoma haematobium (57). An analysis of 22 human liver samples at different fibrosis stages (F0 ~ F4) suggested that IL-17A promotes fibrosis by inducing the expression of IL-6 and other pro-fibrotic markers such as IL-22 and TGF-β1 (58). These findings confirm that IL-17A contributes to liver fibrosis through two primary pathways: (1) IL-17A expressed in the liver interstitium directly activates HSCs to produce large amounts of collagen; (2) IL-17A stimulates endothelial cells and fibroblasts to secrete various cytokines, chemokines, and cell adhesion factors, inhibited ECM degradation, and promotes fibroblasts proliferation. IL-17A-mediated immune responses significantly affect the hepatic microenvironment, advancing liver fibrosis and correlating positively with fibrosis severity (53).

IL-17A plays a key role in the inflammatory pathways associated with liver injury and may serve as a potential adjunctive diagnostic marker for liver fibrosis (59). Since Cirrhosis can progress to Hepatocellular Carcinoma, studies have suggested that the combined measurement of Alpha-Fetoprotein and IL-17 levels in peripheral serum can predict the prognosis of cirrhotic patients (60). In Hepatitis C Virus (HCV)-associated liver fibrosis, elevated serum IL-17A levels are positively correlated with aminotransferases levels, alpha-fetoprotein concentrations, and fibrosis staging scores, indicating that IL-17A could serve as a biomarker for inflammation and fibrosis progression in chronic HCV infection (61). Therapeutically, IL-17A inhibitors have shown promise in preclinical and clinical studies. These inhibitors suppress HSCs activation and collagen production (51). In a mouse model of Bile Duct Ligation (BDL)-induced liver fibrosis, IL-17A antibody therapy reduced hepatocyte necrosis, decreased pro-inflammatory cytokines, and mitigated neutrophil and macrophage infiltration (62). Furthermore, IL-17A antibody therapy ameliorated hepatic fibrosis in a NASH mouse model (63). Interestingly, IL-17A antibody treatment improved liver fibrosis in 10 psoriasis patients (64), highlighting its potential as a therapeutic target for anti-fibrotic therapies.

### Interleukin-18

IL-18, a member of the IL-1 superfamily originally identified as an Interferon-gamma (IFN-γ) inducer, plays a critical role in regulating both innate and adaptive immune responses (65). IL-18 exerts its immunomodulatory effects primarily binding to the Interleukin-18 Receptor (IL-18R) on the cell membrane (66). It is predominantly synthesized and secreted by KCs and monocytes (67).

In mice with NASH-associated liver fibrosis induced by a high-fat diet, Studies have shown that IL-18 promotes the progression of liver fibrosis. Single-cell RNA sequencing data revealed high expression levels of IL-18 and IL-18R1 on mouse HSCs. Treatment of primary mouse HSCs with recombinant IL-18 accelerated their differentiation into myofibroblasts. Furthermore, the activation of HSCs triggered by NLRP3 inflammasome activation was inhibited when IL-18 signaling was blocked by its natural antagonist, Interleukin-18 Binding Protein (68).

A recent study found that, in a mouse model of CCl4-induced liver fibrosis, the Citrus aurantium extract astragalin inhibited the expression of TNF-α, IL-18, and IL-1β mRNAs in the livers of fibrotic mice through the NF-κB/NLRP3 inflammasome pathway, significantly ameliorating liver fibrosis (69). In the mouse model of liver fibrosis induced by thioacetamide, ginsenoside was shown to inhibit the entry of IL-1β and IL-18 into the extracellular matrix by regulating ERRα-P2X7r signaling pathway, thereby reducing inflammatory responses, improving hepatocyte damage, and suppressing liver fibrosis (70). In clinical studies, the expression level of IL-18 in the peripheral serum of patients with hepatitis B-associated cirrhosis complicated by hepatorenal syndrome (HRS) was significantly higher than in patients without this complication. The sensitivity and specificity of IL-18 for predicting HRS were 90.32% and 71.70%, respectively, suggesting that IL-18 could predict the prognosis of patients with hepatitis B-related cirrhosis (71). Additionally, another study suggested a potential association between the IL-18 -137G/C gene variant and the risk of cirrhosis susceptibility (72). These studies indicate that traditional Chinese medicine monomer compounds can improve liver fibrosis by inhibiting IL-18. Furthermore, IL-18 could serve as a therapeutic target and potential biomarker for the prognosis of liver fibrosis. However, extensive clinical and basic research is still required to confirm these findings.

### Interleukin-33

IL-33, a member of the IL-1 cytokine superfamily, is a key regulator in pathological inflammation, immune homeostasis, and fibrosis. IL-33 plays a key role in innate and adaptive immunity, contributes to tissue homeostasis, and responds to environmental stress. It is abundantly expressed in the endothelial and epithelial cells during both homeostasis and inflammation (73). In the liver, IL-33 is primarily expressed in HSCs and KCs (74). Its reporter, Suppression of Tumorigenicity 2 (ST2), also known as Interleukin-1 Receptor-Like 1, is predominantly expressed in tissue-resident immune cells (75). Upon cellular damage, IL-33 binds to ST2, stimulating immune cell activity (76). The IL-33/ST2 signaling axis is pivotal in liver fibrosis, balancing inflammation with tissue regeneration, wound healing, and tissue repair.

IL-33 has been found to correlate positively with collagen expression, with HSCs being the primary source of IL-33 in fibrotic liver (77). Protein and mRNA levels of IL-33 and ST2 are elevated in fibrotic livers of both mice and humans, with these levels significantly increasing as fibrosis progresses (78, 79). In a mouse model of hepatic fibrosis, IL-33 knockdown led to a marked improvement in fibrosis (80). However, another study reported that IL-33 deficiency did not attenuate liver fibrosis in a high-fat diet-induced steatohepatitis model (81). In a schistosome mouse model, both IL-33 and ST2 levels were elevated, suggesting that the IL-33/ST2 axis might serve as a therapeutic target for liver fibrosis (82). However, while IL-33-targeted therapy mitigates high-fat diet-induced hepatic steatosis, it can exacerbate liver fibrosis through ST2 signaling (79).

Numerous studies have confirmed that the Interleukin-33/T helper 2 cells (IL-33/Th2) axis contributes to liver fibrosis (83). Group 2 innate lymphoid cells (ILC2s), a novel type of innate immune cell in the lymphocyte lineage, are important for liver immune homeostasis and secrete pro-fibrotic cytokines that mediate fibrogenesis. IL-33 attracts ILC2s and activates HSCs by inducing cytokines such as IL-13. Animal studies have shown that ST2 deletion reduces liver injury, inflammatory cell infiltration, and fibrosis. IL-33 activates and aggregates ILC2 cells via ST2 in the liver, and the activated ILC2s secrete IL-13, which in turn activates HSCs through the IL-4Rα-STAT6 transcription factor pathway (80). Furthermore, IL-33 recruits and activates Th2-like CD4+ T cells, which enhance HSCs activation in an Interleukin-13 (IL-13)-dependent manner (84). The IL-33-mediated Th2 immune response promotes HSCs proliferation, TGF-β synthesis, and collagen deposition, with overexpression of IL-33 inducing liver fibrosis. This suggests that IL-33 exerts its pro-fibrotic effects primarily through IL-13 (77).

Regulatory T cells (Tregs), which provide negative feedback regulation of immune responses, are also influenced by IL-33. Studies have shown that Tregs activation by IL-33 is dependent on MyD88. IL-33 binding to ST2 on Tregs activates MyD88, resulting in the expansion of Foxp3+ Tregs *in vivo* (85). In liver fibrosis, IL-33 may play a dual regulatory role through ST2+Treg. On one hand, activated Tregs can promote fibrogenesis. In chronic hepatitis C, Tregs accumulate at fibrotic sites and secrete interleukin-8, which acts on HSCs to upregulation fibrosis-related factors and collagen (86). Additionally, Tregs may adopt a Th2-like role, with IL-33 inducing ST2+ Foxp3+ Tregs to promote fibrosis (87). On the other hand, Tregs exert anti-fibrotic effects via IL-10 secretion. In a BDL-induced liver fibrosis model, Treg inhibition resulted in reduced IL-10 expression, increased fibrosis, and greater inflammatory cell infiltration (88).

Genetic studies have identified associations between IL-33 gene variants and susceptibility to HBV-related cirrhosis. IL33-rs4742170C, rs1048274G, and rs10975519C variants may serve as potential biomarkers for diagnosing HBV-associated cirrhosis (89). In HCV-associated fibrosis, serum IL-33 levels correlate with fibrosis stage and viral load, suggesting that IL-33 could be a biomarker for disease progression (90). Recent research (91) has identified a new chemokine, PSMP (PC3-secreted microprotein), whose receptor is C-C Chemokine Receptor 2 (CCR2). PSMP promotes hepatic fibrosis by polarizing macrophages and directly activating HSCs via CCR2. IL-33, as a damage-associated molecular pattern (DAMP), enhances PSMP production, highlighting its critical role in hepatic fibrosis progression (92). In BDL-induced hepatic fibrosis models, injury-induced release of endogenous IL-33 triggers inflammation and fibrosis. However, acute massive liver injury sees IL-33 activating tissue-protective mechanisms, whereas in chronic injury, it promotes fibrosis (77). Melatonin has demonstrated protective effects in hepatic ischemia-reperfusion injury by inhibiting oxidative stress and apoptosis through the IL-33 signaling pathway (93). The role of IL-33 in liver fibrosis warrants further exploration and holds potential as a therapeutic target for fibrosis treatment.

### Interleukin-36

IL-36 is a member of the IL-1 superfamily. It Regulates immune cell responses, and activates fibroblasts. IL-36 consists of three receptor agonists- IL-36α, IL-36β, and IL-36γ-as well as the IL-36 receptor antagonist (IL-36Ra). Initially, IL-36 was also referred to as IL-1F6, IL-1F8, among another names. The receptors for IL-36 are primarily IL-36 receptor alpha (IL-36Rα) and IL-1 receptor accessory protein IL-1RAcP, which together form a complex consisting of two subunits (94). IL-36 cytokines are expressed in various cell types, including keratinocytes, monocytes, and dendritic cells (DCs). In the liver, IL-36 is predominantly expressed in macrophages, DCs, and endothelial cells. IL-36α is expressed during embryonic development and is highly enriched in epithelial cells, monocytes, B cells, and T cells (95, 96). IL-36β is also expressed in epithelial cells and is regulated by epidermal growth factor (97). IL-36γ is expressed in stimulated esophageal keratinocytes and squamous epithelium cells (96, 98). IL-36 influences inflammatory responses through multiple mechanisms, including inducing the secretion of inflammatory mediators and chemokines and modulating immune cell function.

Research indicates that IL-36 participates in early tissue inflammatory responses via multiple immune pathways (99). In an animal model of liver injury, IL-36 was implicated in the early inflammatory response, as high levels of C-C Motif Chemokine Ligand 20 (CCL20) expression were detected in liver tissues. CCL20 activation promotes liver fibrosis development (100). In a mouse model of acetaminophen-induced liver injury, IL-36γ and CCL20 were both highly expressed. Treatment with IL-36Ra reduced CCL20 expression at both the mRNA and protein levels and alleviated liver injury. This suggests that blocking IL-36 signaling can mitigate liver inflammation by decreasing CCL20 expression and, consequently, may intervene in the progression of liver fibrosis (101).

IL-36 plays an important role in liver fibrosis. Studies have shown that IL-36 exerts pro-fibrotic effects by binding to its receptor. However, no drugs have been developed specifically targeting IL-36 for liver fibrosis treatment. The use of recombinant IL-36Ra or IL-36R blockers represents a potential therapeutic strategy and warrants further investigation.

**Figure 1:**
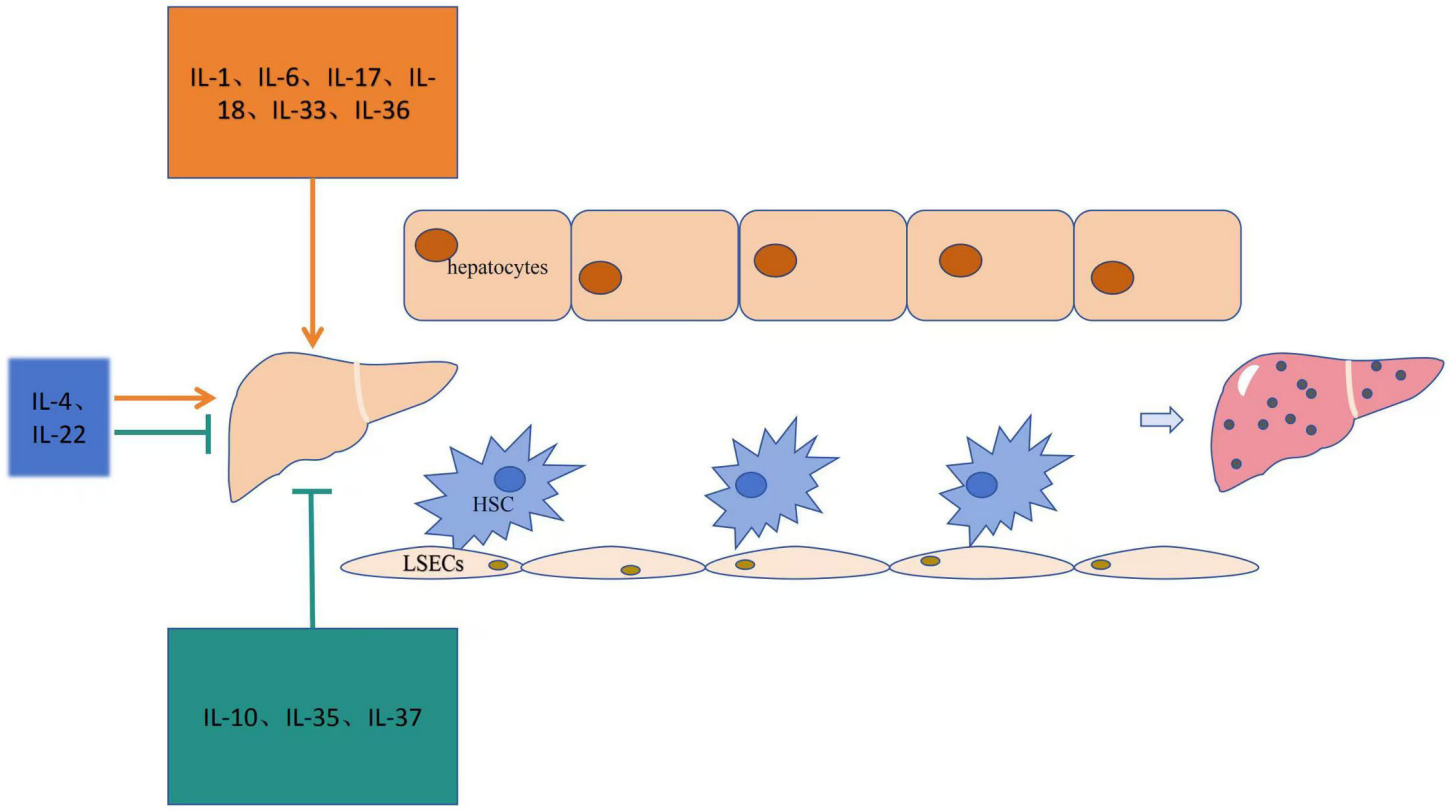
Mechanism of action of the interleukin family in liver fibrosis.

The role of interleukins in promoting liver fibrosis is shown in **Table 1**. **Table 2** summarizes the signaling pathways involved in the pro-fibrotic effects of interleukins, and **Figure 1** illustrates the mechanisms through which these pro-fibrotic interleukins act in liver fibrosis.

**Table 1:** Role of interleukins associated with liver fibrosis.

Categorization	The Interleukins	Primary role	Role in liver fibrosis	Bibliography
Interleukins exerting pro-liver fibrosis effects	IL-1	Pro-inflammatory, pro-fibrotic	Promote liver cell death Promotes the release of pro-fibrotic factors TGF-β and PDGF, activation of HSCs, and ECM production.	(9–32)
	IL-6	Pro-inflammatory, pro-fibrotic	Activation of HSCs is positively correlated with the degree of liver inflammation and liver fibrosis	(33–49)
	IL-17	Pro-inflammatory, pro-fibrotic	Promotion of pro-inflammatory cytokines, neutrophil and macrophage infiltration Promote the expression of pro-fibrotic factor receptor to stimulate the activation of HSCs, inhibit the decomposition of ECM, and stimulate the proliferation of fibroblasts.	(50–64)
	IL-18	Pro-inflammatory, pro-fibrotic	Activation of HSCs, accelerated differentiation of HSCs into myofibroblasts promote liver fibrosis	(65–72)
	IL-33	Pro-inflammatory, pro-fibrotic	Attracts type 2 intrinsic lymphocytes and activates HSCs by releasing IL-13. Aggregation of large amounts of Treg to the site of fibrosis caused upregulation of the expression of fibrosis-associated factors and collagen Promotes infiltration and polarization of inflammatory macrophages and production of pro-inflammatory cytokines	(73–93)
	IL-36	Pro-inflammatory, pro-fibrotic	Involved in the immune-inflammatory response in the liver, promotes the expression of the pro-inflammatory chemokine CCL20 aggravates liver inflammation and promotes hepatic fibrosis.	(94–101)
Interleukins exerting anti-liver fibrosis effects	IL-10	Regulatory cytokine, anti-inflammatory	Inhibiting the release of pro-fibrotic factors as well as inflammatory mediators, reducing hepatocyte apoptosis, and inhibiting autophagy in HSCs. Inhibits ECM synthesis and promotes its degradation	(102–118)
	IL-35	Anti-inflammatory, anti-fibrotic	Inhibits the proliferation of CD4 CD25 effector T cells and the differentiation of Th17 cells to attenuate liver inflammation.	(119–128)
	IL-37	Anti-inflammatory, anti-fibrotic	Promotes macrophage polarization from M1-type to M2-type and down-regulates the expression of associated inflammatory chemokines. Limiting lymphocyte, macrophage and KC infiltration into the liver and inhibiting the release of pro-inflammatory and pro-fibrotic cytokines attenuates liver inflammation, inhibits HSCs activation and reduces collagen deposition.	(128–131, 132)
Interleukins that exert bidirectional regulation of liver fibrosis	IL-4	Dual anti-inflammatory and pro-inflammatory effects	Increases collagen synthesis and inhibits cell proliferation. Promoting macrophage polarization towards the M2 phenotype decreases pro-inflammatory cytokine expression and increases expression of the anti-inflammatory cytokine IL-10	(133–139)
	IL-22	Dual anti-inflammatory and pro-inflammatory effects, promoting tissue repair	Down-regulate the level of inflammatory cytokines, inhibit the activation of HSCs, hepatocyte autophagy, and promote the proliferation of liver progenitor cells and tissue repair to play a role in protecting the liver Promotes Th17 recruitment to sites of liver inflammation, exacerbates liver inflammation and promotes hepatic fibrosis progression	(31, 124, 140–150)

**Table 2:** Signaling pathways involved in interleukins in liver fibrosis.

Categorization	Interleukin	Key Receptors	Residence in the liver	Key Signaling Pathways
Interleukin exerting pro-liver fibrosis effects	IL-1	IL-1R1/IL-1RAcP	Kupffer cells, liver endothelial cells, HSCs	MyD88-IRAK-NF-κB
	IL-6	IL-6R/gp130	Hepatocytes, Kupffer cells, HSCs	JAK/STAT3, MAPK (ERK1/2), gp130
	IL-17	IL-17RA/IL-17RC	Th17 cells, hepatocytes, Kupffer cells	NF-κB
	IL-18	IL-18R	Kupffer cells, monocytes	NF-κB/NLRP3, ERRα-P2X7r
	IL-33	ST2 (IL1RL1)	HSCs, Kupffer cells	IL-33/ST2, IL-13/STAT6
	IL-36	IL-36R/IL-1RAcP	Macrophages, dendritic cells, endothelial cells	CCL20-related signaling
Interleukin exerting anti-liver fibrosis effects	IL-10	IL-10R (IL-10R1/IL-10R2)	Hepatocytes, Kupffer cells, regulatory B (Breg) cells, Treg cells	STAT3-related signaling
	IL-35	IL-12Rβ2/IL-27Rα	Regulatory T cells	Smad3/TGF-β
	IL-37	–	Hepatocytes, HSCs, Kupffer cells	AMPK-Smad3
Interleukins that exert bidirectional regulation of liver fibrosis	IL-4	IL-4R	Kupffer cells	PPARγ/STAT3, MMP10
	IL-22	IL-22R1/IL-10R2	CD4 and CD8 T cells, γδT cells, NK cells, innate lymphoid cells (ILCs)	STAT3, PI3K/AKT/mTOR, IL-22BP

## Interleukins exerting anti-liver fibrosis effects

### Interleukin-10

IL-10 is a cytokines with anti-inflammatory and immunosuppressive effects. It reduces immune cell infiltration and inhibits the release of inflammatory factors. The IL-10 receptor (IL-10R) is primarily composed of two subunits: IL-10R1 and IL-10R2 (102). In the liver, IL-10 can be secreted by hepatocytes, KCs, regulatory B (Breg) cells and Treg cells, among other cell types (103). IL-10 promotes the translocation of phosphorylated STAT3 from the cytoplasm to the nucleus by enhancing phosphorylation signal transduction and STAT3 expression, thereby activating STAT3 and inhibiting autophagy (104). It also regulates autophagy in HSC-T6 cells through the STAT3-mTOR-p70S6K axis (105) and inhibits autophagosome formation in HSCs induced by oxidative stress by activating the mTOR-STAT3 pathway (106). This process suppresses the release of inflammatory factors, inhibits immune cell activation, and alleviates liver fibrosis.

IL-10 inhibits immune cell activation, attenuates hepatic inflammatory, and exerts protective effects on liver fibrosis progression (107). It effectively reduces the production of the pro-fibrotic factor TGF-β1, inhibits macrophages synthesis and secretion of TNF-α, downregulate the downstream effector NF-κB, and remodels the ECM (108). During liver fibrosis, IL-10 mitigates fibrosis by suppressing inflammatory cellular immune responses and the production of TGF-β1, TNF-α, and tissue inhibitors of metalloproteinases (107, 109). Additionally, IL-10 inhibits ECM synthesis and promotes its degradation (110, 111).

In a mouse model of liver fibrosis induced by BDL, IL-10 was highly expressed in HSCs (112). Elevated *IL-10* expression in human liver tissues reduces hepatocyte apoptosis and reverses liver fibrosis (113, 114). HSCs may secrete autocrine IL-10 to inhibit collagen synthesis, suppress liver inflammatory responses, and slow the progression of liver fibrosis (115). A study reported that using IL-10-modified bone marrow-derived DCs, where the DCs were immature and infused into CCL4-induced hepatic fibrotic mice, significantly alleviated liver fibrosis. This treatment increased Treg cell expression in the liver while reducing inflammatory cytokines such as Interleukin-12 (IL-12), IL-22, and TNF-α (116). Furthermore, *in vitro* studies showed that IL-10 secretion by immature dendritic cells (imDCs) increased apoptosis and inhibited the proliferation of LX-2 cells. It also downregulated α-SMA mRNA expression and decreased TGF-β1 and Smad3 proteins levels, suggesting that IL-10 secretion by imDCs inhibit LX-2 activation via suppressing of the TGF-β1/Smad3 pathway (117). Recent studies indicate that IL-10 gene intervention enhances the accumulation of NK cells in the liver by improving their immune functions, including activation, cytotoxicity, development, and migration. It also increases the expression of NKG2D, IFN-γ, and CD107a in the liver tissue, thereby alleviating liver fibrosis (118).

The finding provide new theoretical support for the anti-fibrotic effects of IL-10. As a key negative feedback regulator, IL-10 prevents the onset and progression of liver fibrosis by inhibition the release of inflammatory mediator, suppressing NF-κB activity, and acting through other mechanisms to protect the liver. Although the anti-fibrotic properties of IL-10 are well-recognized, its precise mechanisms and clinical application prospects require further in-depth research and exploration.

### Interleukin-35

IL-35 is a newly identified cytokines and a member of the IL-12 cytokine superfamily. It is a heterodimer composed of EBV-inducible gene 3 and IL-12p35, primarily secreted by Treg cells. IL-35 enhances Treg function through positive feedback and plays a critical role in maintaining immune homeostasis within the hepatic microenvironment. The IL-35 receptor (IL-35R) is primarily composed of two subunits: IL-12 receptor beta 2 (IL-12Rβ2) and IL-27 receptor alpha (IL-27Rα) (119). IL-35 inhibits the proliferation of CD4+ CD25+ effector T cells and the differentiation of Th17 cells, thus contributing to immune balance in the liver (120–122).

Serum IL-35 levels are significantly elevated in patients with HBV-associated cirrhosis compared to healthy controls and show a positive correlation with IL-17, IL-22, and IL-33 (123, 124). In the early stage of HBV-associated cirrhosis, the number of Th17 cells increases, and HSCs become activated, secreting pro-inflammatory cytokines such as IL-17 and TGF-β. To mitigate inflammatory damage to hepatocytes, Treg cells secrete IL-35 and IL-10, effectively suppressing Th17 differentiation and IL-17 production in HBV-associated cirrhosis (125). Furthermore, IL-35 knockdown increase the expression levels of IL-17 and IL-22 (126). IL-35 inhibits the binding of TGF-β to its receptor and suppresses the phosphorylation of Smad3, a downstream effector of the TGF-β receptor. This mechanism reduces Th17 differentiation and IL-17 synthesis in patients with HBV-associated cirrhosis (126). Additionally, the expression of IL-35 correlates with the histological grade and severity of PBC. serum IL-35 levels are higher in patients with stage III and IV disease compared to those in stage II. Moreover, IL-35 mRNA and protein levels negatively correlate with the Child-Pugh score for cirrhosis severity (127, 128).

IL-35 shows potential to improve liver fibrosis by inhibiting Th17 differentiation and IL-17 synthesis. However, its precise mechanisms remain unclear, and no anti-fibrotic drugs targeting IL-35 have been developed. Further research is needed to elucidate the specific roles and mechanisms of IL-35 in liver fibrosis.

### Interleukin-37

IIL-37 is an important anti-inflammatory cytokines within the IL-1 family. It plays a crucial role in the prevention and treatment of liver fibrosis. IL-37 is expressed in various immune cells, including NK cells, B cells, T cells, and macrophages (129). In liver tissues, IL-37 is predominantly expressed in hepatocytes and can also be detected in intrahepatic cholangiocytes, HSCs and KCs (130).

IL-37 mitigates liver inflammation and alleviates liver fibrosis by inhibiting the expression of pro-inflammatory cytokines and chemokines in hepatocytes and KCs, reducing neutrophil activity, and acting directly on hepatocytes. Serum IL-37 levels have been found to be higher in patients with cirrhosis compared to healthy controls and positively correlated with cirrhosis stage score. In a BDL-induced mouse model of liver fibrosis, IL-37 overexpression reduced the inflammatory response and HSCs activation (131). In IL-37 transgenic mice with BDL-induced liver fibrosis, reduced expression levels of early liver fibrosis markers, such as C-X-C Motif Chemokine Ligand 2, were observed, along with decreased collagen deposition and liver fibrosis. *In vitro* experiments further demonstrated that IL-37 inhibits IL-1-induced activation of HSCs (131). Additionally, another study found that IL-37 promotes macrophages polarization from the pro-inflammatory M1 type to the anti-inflammatory M2 type and downregulates the expression of inflammatory chemokines, thereby inhibiting liver fibrosis. This mechanism may be associated with AMP-activated Protein Kinase (AMPK) pathway activation induced by Smad3 interaction (132). Recent studies have reported that IL-37 inhibits the activation of KCs and HSCs and interferes with TGF-β signaling, thereby reducing liver fibrosis and inflammation levels (131). Furthermore, IL-37 exerts protective effect against hepatic ischemia/reperfusion injury by decreasing pro-inflammatory cytokines and chemokines produced by hepatocytes and KCs, directly protecting hepatocytes from damage. As a novel anti-inflammatory factor, IL-37 holds promise as a potential therapeutic target for liver fibrosis.

**Table 1** summarizes the role of interleukins in anti-liver fibrosis. **Table 2** outlines the signaling pathways involved in the anti-fibrotic effects of interleukins, and **Figure 1** illustrates the mechanisms through which these interleukins exert their anti-fibrotic effects.

## Interleukins that exert bi-directional regulation of liver fibrosis

### Interleukin-4

IL-4 is a Th2 cytokines that regulates the immune response, including eosinophil recruitment, parasite clearance, and IgE class switching, which can lead to hypersensitivity reaction. Additionally, IL-4 inhibits the activity of Th1 cells, thereby exerting some anti-inflammatory effects (94). It is mainly expressed in KCs in the liver. Its receptor is IL-4R (133). In the liver, IL-4 plays a dual role in liver fibrosis by inducing KCs to transform into multinucleated giant cells, stimulating the proliferation of HSCs, upregulating PPARγ, and regulating macrophage polarization (134).

Studies have shown that IL-4 induces hepatic KCs to transform into multinucleated giant cells and stimulates HSCs proliferation (135). Moreover, IL-4 can act directly on HSCs to promote their proliferation, increase collagen production, and accelerate the progression of liver fibrosis (136, 137). In addition, IL-4 can stimulate macrophage polarization toward the M2 phenotype. During this process, PPARγ binds to the matrix metallopeptidase 10 (MMP10) promoter, upregulating MMP10 expression. This mechanism further activates the downstream STAT3 signaling pathway, inducing M2 macrophage polarization. As a result, pro-inflammatory cytokines such as IL-1β and TNF-α are downregulated, while the anti-inflammatory cytokines IL-10 is upregulated. These changes contribute to the amelioration of hepatic steatosis and fibrosis (138). Furthermore, melatonin has been shown to attenuate thioacetamide-induced liver fibrosis in male rats by modulating IL-6, IL-4, apoptosis, and urokinase-type plasminogen activator receptor-related protein/Endo180 expression (139). These findings suggest that IL-4 could serve as a potential therapeutic target for liver fibrosis.

### Interleukin-22

IL-22,also known as IL-10-related T cell-derived inducer (IL-T1F), belongs to the IL-10 family. IL-22 is produced by a variety of immune cells, including CD4+ and CD8+ T cells, γδT cells, NK cells, and ILCs (140). It exerts its biological function by binding to the heterodimeric membrane receptor complex IL-22R1/IL-10R2, which is specifically expressed on the surface of tissues such as the skin, kidney, and liver. The IL-22/IL-22R1/IL-22R2 complex primarily activates the downstream JAK-STAT signaling pathway, mainly involving STAT3. IL-22 can also activate the p38 kinase, c-jun N-terminal kinase (JNK), and ERK1/2 pathways (141).

IL-22 has been found to have anti-liver fibrosis effects, IL-22 overexpression attenuates hepatic fibrosis by inhibiting the activation of HSCs and downregulating inflammatory cytokines (142). In a CCl4-induced mouse model of liver fibrosis, IL-22 attenuated fibrosis by regulating cell polarization, inhibiting the STAT3/Erk/Akt pathway, and increasing the M2/M1-KCs ratio of KCs (143). In addition, in the CCL4 mouse model of liver fibrosis, it was found that HSCs could expressed IL-22 receptor 1 in large quantities. IL-22 activated the STAT3 signaling pathway by binding to its receptor, which in turn induced senescence in HSCs and attenuated liver fibrosis (144). In the BDL-induced mouse model of liver fibrosis, IL-22 was shown to increase collagen type I expression while significantly reducing α-SMA mRNA expression, suggesting its antifibrotic effect (53). In the schistosome-induced mouse liver fibrosis model, liposomal IL-22 improved fibrosis via the miR-let7a/STAT3 signaling pathway (145). In another study, inflammatory cells in CHB patients, which promoted hepatic progenitor cell proliferation and tissue repair through the STAT3 signaling pathway, suggesting that IL-22 plays a protective role in liver repair (146). It has also been found that IL-22 binding protein (IL-22BP), an inhibitor of IL-22, can aggravate fibrosis and cirrhosis in chronic HCV-infected patients (147). In an alcoholic mouse model of liver fibrosis, IL-22 ameliorated fibrosis partly by inhibiting hepatocyte autophagy and the PI3K/AKT/mTOR pathway (148).

However, IL-22 has also been reported to exert pro-liver fibrosis effects. In HBV-infected cirrhotic patients and an HBV transgenic mouse model of chronic liver inflammation and fibrosis, IL-22 induced the recruitment of Th17 cells to the sites of liver inflammation. It promoted the production of more IL-22, creating a positive feedback loop that exacerbated chronic inflammation and fibrosis. In a study involving 74 CHB patients, 36 hepatitis B cirrhosis patients, and 10 healthy controls, IL-22 levels positively correlated with the degree of liver fibrosis. When IL-22 was applied to stimulate HSCs *in vitro*, it produced chemokines that attracted Th17 cells, accelerating liver inflammation and fibrosis (124). IL-22 may lose its protective effect in the presence of IL-17 and even have pathogenicity (149). Studies have reported that an increase in IL-22-positive cells in the liver of HCV-infected patients correlated with fibrosis staging scores and clinical progression from chronic hepatitis to cirrhosis. *In vitro* experiments showed that IL-22 increased α-SMA expression and collagen production by inhibiting apoptosis and promoting proliferation of LX-2 cells (150).

Angelica sinensis polysaccharide (ASP) has hepatoprotective effects. In a CCl4-induced mouse model of liver fibrosis, ASP promoted IL-22 secretion, inhibited HSCs activation, and effectively attenuates fibrosis through the IL-22/STAT3 pathway (31). The balance between anti-inflammatory and pro-inflammatory effects of IL-22 may determine its role in liver fibrosis, and its exact mechanisms require further investigation. The relationship between IL-22 and liver fibrosis remains controversial. These seemingly contradictory results may be due to differences in disease models, degrees of liver injury, immune microenvironment, and cytokines interactions, The dual anti-inflammatory and pro-inflammatory roles of IL-22 in fibrosis caused by HBV and HCV infections warrant in-depth exploration to better understand its role in liver fibrosis.

**Table 1** illustrates the bidirectional regulatory effects of interleukins on liver fibrosis. **Table 2** shows the signaling pathways related to interleukins involved in liver fibrosis, and **Figure 1** explains the mechanisms through which these interleukins act in liver fibrosis.

## Conclusions and perspectives

Liver fibrosis is a dynamic pathological process involving multiple factors and pathways, resulting from intricate cellular crosstalk. In recent years, significant progress has been made in understanding the pathogenesis and exploring treatments for liver fibrosis. However, effective anti-fibrotic therapies are still lacking in clinical practice. The interleukin family of cytokines plays a pivotal role in the initiation and regulation of inflammation, as well as in innate and adaptive immunity, by activating complex cascades involving cytokines, chemokines, adhesion molecules, and lipid mediators. In the context of liver fibrosis, many interleukins, such as IL-1, IL-6, IL-17, IL-18, IL-33, and IL-36, exhibit pro-fibrotic effects. These cytokines exacerbate fibrosis by inducing the infiltration monocytes/macrophages infiltration into liver tissues, upregulating pro-inflammatory and pro-fibrotic cytokines, and promoting the proliferation and activation of HSCs. Conversely, certain interleukins, including IL-10, IL-35 and IL-37 have protective effects against liver fibrosis. These cytokines suppress the release of pro-inflammatory mediators, inhibit fibrosis-related pathways and ECM synthesis, and promote ECM degradation.

Moreover, interleukins such as IL-4 and IL-22 exhibit dual roles, functioning as both anti-inflammatory and pro-inflammatory mediators in liver fibrosis, warranting further investigation. Developing interleukin-targeting therapies, either inhibitors or agonists, holds promise as a potential treatment strategy. However, current research is predominantly at the cellular and animal model levels. Further studies are needed to elucidate the underlying mechanisms and evaluate the clinical applicability of these therapies. Emerging technologies and advancements in interleukin-based treatments are anticipated to play a positive role in managing liver fibrosis in the future.
